# Mitis Group Streptococci Express Variable Pilus Islet 2 Pili

**DOI:** 10.1371/journal.pone.0025124

**Published:** 2011-09-22

**Authors:** Dorothea Zähner, Ashish R. Gandhi, Hong Yi, David S. Stephens

**Affiliations:** 1 Division of Infectious Diseases, Department of Medicine, Atlanta, Georgia, United States of America; 2 Department of Microbiology and Immunology, Emory University School of Medicine, Atlanta, Georgia, United States of America; 3 Robert P. Apkarian Integrated Electron Microscopy Core, Emory University, Atlanta, Georgia, United States of America; 4 Department of Veterans Affairs Medical Center (Atlanta), Decatur, Georgia, United States of America; Charité-University Medicine Berlin, Germany

## Abstract

**Background:**

*Streptococcus oralis, Streptococcus mitis,* and *Streptococcus sanguinis* are members of the Mitis group of streptococci and agents of oral biofilm, dental plaque and infective endocarditis, disease processes that involve bacteria-bacteria and bacteria-host interactions. Their close relative, the human pathogen *S. pneumoniae* uses pilus-islet 2 (PI-2)-encoded pili to facilitate adhesion to eukaryotic cells.

**Methodology/Principal Findings:**

PI-2 pilus-encoding genetic islets were identified in S. oralis, S. mitis, and S. sanguinis, but were absent from other isolates of these species. The PI-2 islets resembled the genetic organization of the PI-2 islet of S. pneumoniae, but differed in the genes encoding the structural pilus proteins PitA and PitB. Two and three variants of pitA (a pseudogene in S. pneumoniae) and pitB, respectively, were identified that showed ≈20% difference in nucleotide as well as corresponding protein sequence. Species-independent combinations of pitA and pitB variants indicated prior intra- and interspecies horizontal gene transfer events. Polyclonal antisera developed against PitA and PitB of S. oralis type strain ATCC35037 revealed that PI-2 pili in oral streptococci were composed of PitA and PitB. Electronmicrographs showed pilus structures radiating >700 nm from the bacterial surface in the wild type strain, but not in an isogenic PI-2 deletion mutant. Anti-PitB-antiserum only reacted with pili containing the same PitB variant, whereas anti-PitA antiserum was cross-reactive with the other PitA variant. Electronic multilocus sequence analysis revealed that all PI-2-encoding oral streptococci were closely-related and cluster with non-PI-2-encoding S. oralis strains.

**Conclusions/Significance:**

This is the first identification of PI-2 pili in Mitis group oral streptococci. The findings provide a striking example of intra- and interspecies horizontal gene transfer. The PI-2 pilus diversity provides a possible key to link strain-specific bacterial interactions and/or tissue tropisms with pathogenic traits in the Mitis group streptococci.

## Introduction

Oral streptococci of the Mitis group streptococci [1], [2], such as *Streptococcus mitis*, *Streptococcus oralis*, and *Streptococcus sanguinis* are close relatives of the important human pathogen *Streptococcus pneumoniae*. They are members of the normal human oral flora, but in contrast to *S. pneumoniae* only occasionally cause acute or chronic disease as opportunistic human pathogens. But as early colonizers in the development of oral biofilms (for review see [3]), they are associated with gingivitis and caries and occasionally cause subacute infective endocarditis [4]. They share with *S. pneumoniae* the ability to develop natural competence for genetic transformation [5]; and extensive horizontal gene transfer, including virulence genes, has been documented between these species [1], [6], [7], [8]. Hence the genetic diversity within these species results in strain-specific capabilities to form intra- and inter-species biofilms, to interact with salivary components or to bind platelets, all of which are factors important in host colonization and disease development.

Pili, fimbrial extensions on the surface of bacteria have recently been identified in many Gram-positive species, including streptococci [9], [10], [11], [12], [13], enterococci [14], [15], bacilli [16], lactobacilli [17], and actinomyces [18], [19], and they have diverse, and often unresolved roles in environmental interactions or pathogenesis [20]. These pili are typically encoded by gene clusters, sometimes called pilus islets [21], that contain in addition the genes required for pilus biosynthesis [22]. Typically, these gene clusters encode a pilus backbone protein and one or two accessory pilus proteins, which in some pili are either located at the tip of the pilus [23], [24], and may serve as adhesin [24], or at the base of the pilus, acting as a linker between the pilus backbone polymer and the cell wall [24], [25], [26], [27]. All Gram-positive pilus proteins share a C-terminal LPXTG-motif or a variant thereof, as part of a cell wall sorting signal (CWSS). This CWSS is a typical feature of Gram-positive surface proteins that are attached to the cell wall [28], [29]. In addition, at least one sortase enzyme is encoded in the pilus gene cluster. This sortase polymerizes pilus proteins to form the pilus structure [30]. Attachment of the pilus structure to the peptidoglycan precursor of the cell wall is usually performed by the housekeeping sortase, mostly referred to as sortase A [31], which in addition attaches other LPXTG-motif containing surface proteins to the cell surface and is encoded outside the pilus gene cluster. The surface exposure of Gram-positive pili has made them attractive vaccine candidates [32].

In Mitis group streptococci, pili have been identified in *S. sanguinis* and *S. pneumoniae* [9], [13]. *S. pneumoniae* can harbor two different types of pilus islets, the *rlrA* pilus islet, also called pilus islet 1 (PI-1) [9] and the type two pilus islet, named pilus islet 2 (PI-2) [21]. The pilus gene cluster in *S. sanguinis* resembles the PI-1 pilus islet in *S. pneumoniae* [13]. The two pilus islets in *S. pneumoniae* encode two antigenically different pili [21]. The pneumococcal PI-2 islet has been shown to be present in about 16 to 21% of pneumococcal isolates [21], [33]. The pneumococcal PI-2 islet is unusual since it encodes a pilus formed solely by a pilus backbone protein, PitB [21]. A second gene, *pitA*, encodes signature motifs of a Gram-positive surface protein, i.e. an N-terminal signal sequence and a C-terminal CWSS, but it is a pseudogene due to a stop-codon causing its premature termination. The PitB-based pilus has been shown to be present as a single structure on the bacterial surface, in contrast to the typical multitude of pilus structures per bacterial cells observed for other Gram-positive bacteria [21], [22]. An additional unusual feature of the pneumococcal PI-2 islet is that of the two encoded sortase genes, *srtG1* and *srtG2*, the latter is a pseudogene in most of the analyzed PI-2 gene clusters [21]. The pneumococcal PI-2 islet shows an unusual high sequence conservation of >99.9% identity over the entire 6.8 kb region among all PI-2 containing pneumococcal isolates, and is inserted in all analyzed strains in the same insertion site, located between the *pepT* and *hemH* genes [21], [33]. A PI-2-pilitated pneumococcal strain has been shown to mediate host cell adhesion in a pilus-dependent manner [21].

We report the first identification of PI-2 pili in *S. oralis* and *S. mitis*, with several strains expressing pili that, in addition to the pilus backbone protein, have a putative adhesin protein attached to the pilus backbone structure. Genetic variability between genes encoding the PI-2 pilus proteins showed that the PI-2 islet in oral streptococci was subject to horizontal gene transfer between these species. Further, by immunogold electron microscopy (EM), pili were found to share the morphology of the pneumococcal PI-2 pili. The sequence variation in PitB was shown to result in antigenic variation. The variability of the pilus proteins within and beyond the species suggests a role for PI-2 pili in the strain-specific tissue tropism of the Mitis group of streptococci.

## Results

### PI-2 pilus islets in oral Mitis group streptococci

**Figure 1:**
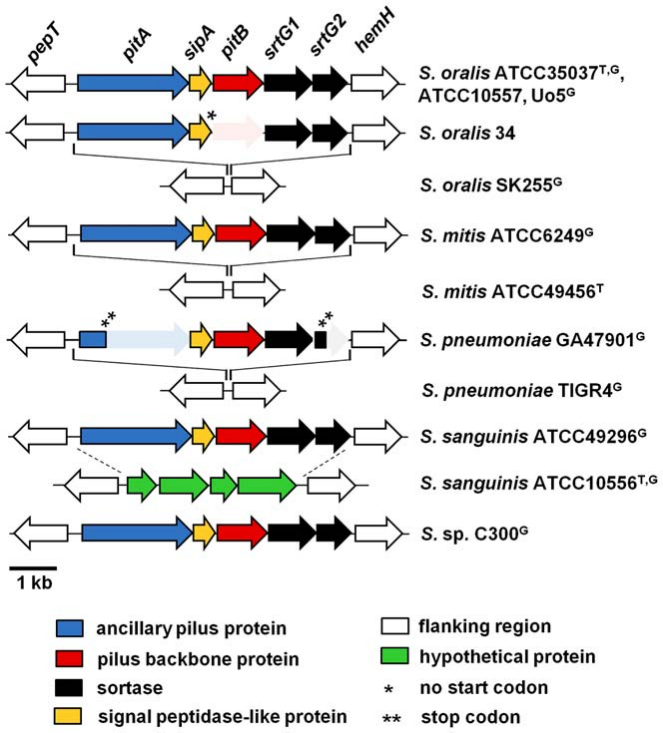
Schematic representation of PI-2 pilus-encoding regions in Mitis group streptococci. Type strains (^T^) and strains from genomic sequencing projects (^G^) are indicated. GenBank accession numbers: *S. oralis* ATCC35037: AEDW01000020; *S. oralis* Uo5: FR720602; *S. oralis* SK255: AFNM00000000; *S. mitis* ATCC6249: AEEN01000012; *S. pneumoniae* GA47901: AFGR00000000*; S. pneumoniae* TIGR4: AE005672; *S. sanguinis* ATCC49296: AEPO01000013; *S. sanguinis* ATCC10556: AFAZ00000000; *S.* sp. C300: ACRJ00000000. Shaded areas indicate pseudogenes (one asterisk indicates a mutation in the start codon and two asterisks indicate the position of a stop-codon).

In a search for pilus-encoding islets that resemble PI-2 of *S. pneumoniae*, the publicly available genomic sequences of oral Mitis group streptococci and selected additional strains (Table 1), isolates that have frequently been used as reference strains in studies of oral streptococci, were examined. PI-2 pilus islets were identified in genomic sequencing projects of the *S. oralis* type strain ATCC35037 [34], *S. mitis* ATCC6249, *S. sanguinis* ATCC49296, and *S.* sp. C300 (all part of the “The NIH human microbiome project” [http://nihroadmap.nih.gov/hmp]), and *S. oralis* Uo5 [35] (Figure 1). In addition, PI-2 pilus islets were identified by PCR in *S. oralis* strain ATCC10557 and strain 34 (both formerly classified as *S. sanguis* [34], [36] (Figure 1). ATCC10557 is an isolate from a patient with endocarditis often used as a *S. oralis* reference strain in adhesion and disease-associated studies [37], and strain 34 is most commonly used in studies of experimental biofilm formation involving *S. oralis* [19]. After initial identification of the PI-2 islet in ATCC10557 and 34 by PCR, the entire DNA sequence of the PI-2 genetic islets was determined. In all seven strains (Figure 1) the PI-2 islet encompassed 6.8 kb and was inserted between the *pepT* and *hemH* genes, consistent with the insertion site of the PI-2 islet in *S. pneumoniae* [21]. In all seven isolates the PI-2 islet encoded the genes for the same five proteins: for two putative pilus proteins, named according to the pneumococcal PI-2 islet, *pitA* and *pitB,* a signal peptidase like-protein, *sipA* [21], [38]; and two putative sortases, *srtG1* and *srtG2*, respectively (Figure 1). The overall similarity between the PI-2 nucleotide sequences of the seven strains ranged from 87% to 94% identity, and their GC-content was 37.8 to 38.4%, which is lower than the average GC-content of the available genomic sequences of the hosting *S. oralis*, *S. mitis*, *S. sanguinis* strains, and *S.* sp. C300, respectively (41%), but closer to *S. pneumoniae* strains (39%), suggesting that the PI-2 islet may have evolved in a different species, with slightly lower GC-content.

**Table 1:** Streptococcal strains used in this study.

Strain	Relevant genotypic features	Source/alternative name/reference
*S. oralis* ATCC35037	PI-2	ATCC^a^; NTCT11427, SK23; type strain
ATCC35037ΔPI2	ATCC 35037-derivative, but PI2 ::km	This study
*S. oralis* ATCC10557	PI-2	ATCC; SK2
ATCC10557ΔPI2	ATCC 10557-derivative, but PI2 ::km	This study
*S. oralis* 34	PI-2	[36]
*S. mitis* ATCC6249	PI-2	ATCC
*S. sanguinis* ATCC49296	PI-2	ATCC
*S. pneumoniae* GA47901	PI-2	[33]
*S. pseudopneumoniae* BAA-960		ATCC; CDC-SS-1757; type strain
*S. gordonii* ATCC10558		ATCC; type strain

a American Type Culture Collection.

The *pitA* gene, which is a pseudogene in the *S. pneumoniae* PI-2 pilus islet, was the first gene of the oral streptococci PI-2 islet (Figure 1) and was predicted to encode in all seven streptococcal strains a functional surface protein, with a typical N-terminal signal peptide and a LPXTG-like motif (VPETG) in the C-terminal CWSS. In addition, PitA in all five strains was predicted to contain a von Willebrand factor type A (vWA) domain [39], characteristic of many eukaryotic extracellular-matrix binding proteins, such as α2β1 integrin, an important human receptor for collagen [40], [41]. The vWA domain resided in the N-terminal region of PitA (amino acid residues 77 to 355 in the PitA sequence of ATCC35037), and contained the characteristic MIDAS motif (Asp85-X-Ser87-X-Ser89 and Asp249) required for metal-conjugation [41], [42]. vWA domains have been identified in several Gram-positive pilus proteins that act as adhesins [17], [27], [43], suggesting a similar role for PitA.

The *pitB* genes in six of the seven strains (exception *S. oralis* 34) encoded a protein with a non-canonical LPXTG-like motif (VTPTG) in the CWSS. The same motif is also present in PitB of *S. pneumoniae* [21]. In addition, amino acid residues that have been shown for pneumococcal PitB to form two intramolecular isopeptide bonds, each formed between a Lys and Asn residue [44] were conserved in all four alleles (Figure S1). Additional motifs described for some Gram-positive pilus backbone proteins, i.e. the pilin motif and the E-box-motif [30], were missing in PitB of the oral streptococci, consistent with their absence in pneumococcal PitB [21]. In strain *S. oralis* 34, the start codon was missing and alternative start codons were not apparent, suggesting that *pitB* was not transcribed in this strain (Figure 1).

The three additional proteins encoded in PI-2, SipA, which has been shown to be essential in pilus biosynthesis in *S. pneumoniae* pilus islet PI-2 and in some pilus gene clusters in group A streptococci [21], [38], and the two sortases SrtG1 and SrtG2, were all predicted to be functional in all strains. In particular, the *srtG2* genes had the typical length and encoded the conserved active-site cysteines required for sortase activity [21], [45], as opposed to the *srtG2* pseudogenes in *S. pneumoniae* strains. Taken together, with the exception of *S. oralis* 34, the presence of the PI-2 pilus islet in *S. oralis* ATCC35037 and ATCC10057, *S. mitis* ATCC6249, *S. sanguinis* 49296, and *S.* sp. C300 suggested the presence of functional pili, consisting of the putative backbone protein PitB and the accessory PitA protein, on the surface of these strains.

### Sequence variation in streptococcal PI-2 islets and pilus proteins

**Figure 2:**
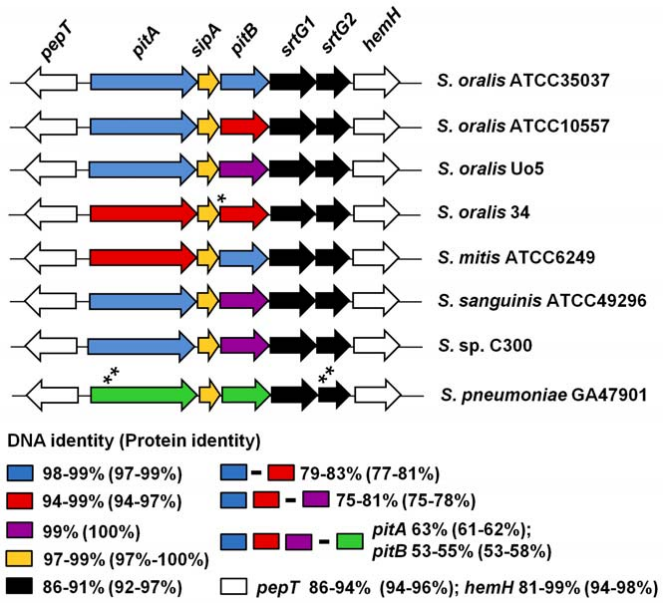
Conservation of genes and proteins in the PI-2 pilus-encoding regions of Mitis group streptococci. Sequence identity within the same variants and between different variants is indicated. GenBank accession numbers are as provided for figure 1. An asterisk indicates a mutation in the start codon; two asterisks the position of a stop-codon.

The PI-2 pilus gene cluster in pneumococcal strains shows an extremely high level of sequence conservation (>99%) throughout the entire 6.8 kb region [2], [33]. In contrast, a comparison of the PI-2 pilus islets of the oral streptococci revealed substantial sequence variation between strains. The DNA sequence variation was highest in the genes encoding the pilus proteins *pitA* and *pitB* (Figure 2). Two variants of *pitA* genes and three variants of *pitB* genes were observed, which differed by approximately 20% in DNA sequence (Figure 2). Surprisingly, the presence of a particular *pitA* variant did not correlate with a particular *pitB* variant even in strains of the same species, such as PitB in ATCC35037 and ATCC10557. Even more striking, gene variants differed between strains of the same species, but the same variants were present in strains of different species, e.g. PitB in *S. oralis* ATCC35037 and *S. mitis* ATCC6249 (Figure 2). A sequence alignment revealed that the different gene variants begin to diverge at the borders to the encoded mature proteins, i.e. immediately after the N-terminal signal peptide and before the C-terminal CWSS signal, resulting in the highest level of protein variation within the region encoding the mature proteins (Figure S2). The past recombination events leading to this diversity may have actually occurred at the border or within the highly conserved *sipA* gene and the flanking *pepT* and sortase genes (Figure 2). The DNA sequence variation between the different *pitA* and *pitB* variants translated into approximately 20% difference in the corresponding protein sequences (Figure 2). The structural genes of the two pilus proteins appear to be exchangeable independently from each other between the strains and species, suggesting the presence of strain-specific pilus variants on the surface of Mitis group streptococci.

### PI-2 pili on the surface of oral streptococci

**Figure 3:**
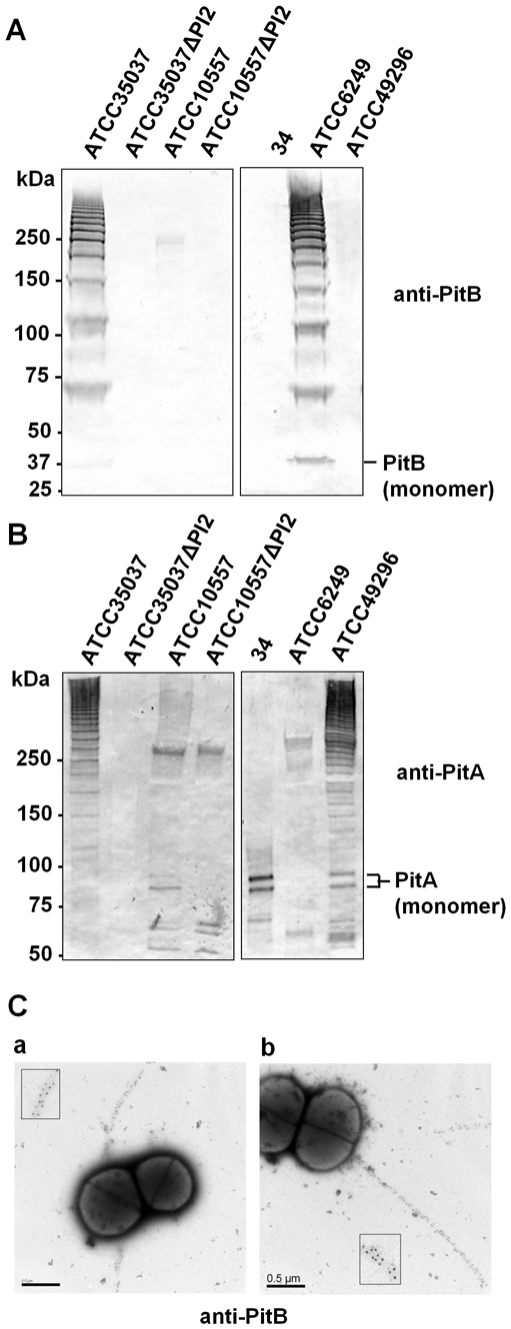
Detection of PI-2 pili on the surface of Mitis group streptococci. Western blots with cell wall extracts of the indicated strains detected with anti-PitB antiserum (A) or anti-PitA antiserum (B). Both antisera were developed against the respective *S. oralis* ATCC35037 proteins. The positions of the PitB and PitA monomers are indicated. (C) Electron micrographs of immmunogold-labeled *S. oralis* ATCC35037 (a) and *S. mitis* ATCC6249 (b) using anti-PitB antiserum. Scale bars 0.5 µm.

To determine whether PI-2 pilus islet-containing strains expose pili on the cell surface, cell wall extracts of strains representing the five different pilus variants, i.e. ATCC35037, ATCC10557, 34, ATCC6249, and ATCC49296 (which encodes pili identical to Uo5 and C300), were prepared and analyzed for the presence of pilus protein-containing polymers by Western blots. Gram positive pilus polymers produce a characteristic high molecular weight (HMW) banding pattern in Western blots developed with an antibody against a pilus protein [10], [11]. Anti-PitB and anti-PitA polyclonal antisera were developed against the ATCC35037 proteins, and PI-2 deletion mutants were constructed in ATCC35037 and ATCC10557 to verify the specificity of the anti-PitB and anti-PitA polyclonal antisera for PI-2-encoded proteins. A Western blot using anti-PitB antiserum revealed HMW bands in cell wall extracts of the ATCC35037 wild type strain (Figure 3A). Corresponding bands were not detected in cell wall extracts of the isogenic PI-2 deletion mutant (Figure 3A), which confirmed the HMW bands detected in the wild type strain were encoded in the PI-2 islet, and that the anti-PitB antiserum was specific for a PI-2 encoded protein. A similar HMW banding pattern was detected in cell wall extracts of *S. mitis* ATCC6249 (Figure 3A), the strain that encoded the same PitB variant as ATCC35037. However, no HMW banding pattern or band corresponding to the molecular weight of the PitB monomer (37 kDa) was detected in cell wall extracts of ATCC10557, its PI-2 deletion mutant, *S. oralis* 34 or *S. sanguinis* ATCC49296. This indicated that either these strains did not express the PitB protein or the anti-PitB antiserum was not cross-reactive with the PitB variants expressed in these strains (Figure 3A). In addition, PitB antiserum was not cross-reactive with PitB of *S. pneumoniae,* and polyclonal antiserum developed against PitB of *S. pneumoniae* [33] was not reactive with any of the identified PitB variants in oral streptococci (data not shown).

To determine whether PitA was present in pilus structures, the cell wall extracts of the five strains and the two PI-2 deletion mutants were examined for HMW bands with the polyclonal anti-PitA antiserum (Figure 3B). HMW banding patterns were observed in cell wall extracts of *S. oralis* ATCC35037, ATCC10557, and *S. sanguinis* ATCC49296 (Figure 3B). The HMW patterns were absent from both isogenic PI-2 deletion mutants, which confirmed the specificity of the anti-PitA antiserum for a PI-2 encoded protein in both strains. In addition, the anti-PitA antiserum reacted with two bands corresponding to the approximate molecular weight of the PitA monomer (88 kDa) in the ATCC10557 wild type strain that were absent in the isogenic deletion mutant (Figure 3B). The two bands suggested the presence of two differently processed species of PitA monomers or a partial instability of the PitA monomer. The HMW banding pattern in ATCC10557 was faint compared to ATCC35037 and ATCC49296, and faint relative to the two bands of the PitA monomer in this strain, suggesting that pilus polymerization occurred in this strain, but was less efficient than in the other two strains (Figure 3B). Detection of HMW bands in ATCC10557 and ATCC49296 with anti-PitA but not with anti-PitB antiserum strongly suggested that a PitB pilus backbone was formed in these strains, but that the PitB variants encoded in these strains eluded detection by the anti-PitB antiserum. HMW bands were not detected in *S. oralis* 34 or *S. mitis* ATCC6249. However, in *S. oralis* 34 two bands that corresponded to the molecular weight of the PitA monomer (88 kDa) were detected, demonstrating that PitA was expressed, but not polymerized (Figure 3B). Since *pitB* is a pseudogene in *S. oralis* 34, this finding was consistent with PitB being the supposed pilus backbone protein of PI-2 pili, as has been shown for the *S. pneumoniae* PI-2 pilus, and PitA an ancillary pilus protein. However, detection of PitA in *S. oralis* 34 also demonstrated that anti-PitA antiserum was able to react with this PitA variant, which is about 20% different from PitA of ATCC35037. Since PitA of *S. oralis* 34 and *S. mitis* ATCC6249 are very similar (>94%), the lack of detection of HMW bands or PitA monomer in the latter strain suggests that PitA may not be expressed in ATCC6249. Taken together, these findings demonstrated the formation of pilus polymers in *S. oralis* ATCC35037, *S. oralis* ATCC10557, *S. mitis* 6249 and *S. sanguinis* ATCC49296. In addition, the results confirmed that PitA, at least in ATCC35037, ATCC10557 and ATCC49296, was part of PI-2 pilus polymers. The PitB variants were antigenically different from each other, whereas the PitA variants showed some cross-reactivity with the used polyclonal antiserum.

To confirm and evaluate the morphology of pilus structures on the surface of the streptococcal isolates that encode PI-2 islets, bacteria were analyzed by EM. Immunogold-labeled anti-PitB-antiserum and anti-PitA-antiserum were used to study the wild type strains and deletion mutants. Anti-PitB-antiserum reacted with pili on the surface of *S. oralis* strain ATCC35037 and *S. mitis* ATCC6249 (Figure 3C). The label was distributed evenly along the pilus structures, which appeared mostly as single pilus per bacterium. The pilus length ranged between 0.7–2.5 µm. Consistent with the results obtained in Western blots, anti-PitB antiserum failed to identify pili on the surface of *S. oralis* ATCC10557 and 34, as well as *S. sanguinis* ATCC49296 (data not shown). Anti-PitA antiserum revealed no pilus structures on the surface of any of the tested strains (data not shown), suggesting that PitA was not present along the pilus backbone. Pilus structures were not visible by negative staining alone and other defined surface structures were not discernible (not shown).

Taken together, pilus structures were observed on the surface of *S. oralis* strain ATCC35037 and *S. mitis* ATCC6249. Strong evidence for the presence of PI-2 pili was obtained for *S. oralis* ATCC10557 and *S. sanguinis* strain ATCC49296 in Western blots using anti-PitA antiserum. The anti-PitB antisera lacked cross-reactivity with pilus protein variants different from ATCC35037.

### Phylogenetic relationship of PI-2-containing isolates and conservation of the PI-2 insertion site in Mitis group streptococci

**Figure 4:**
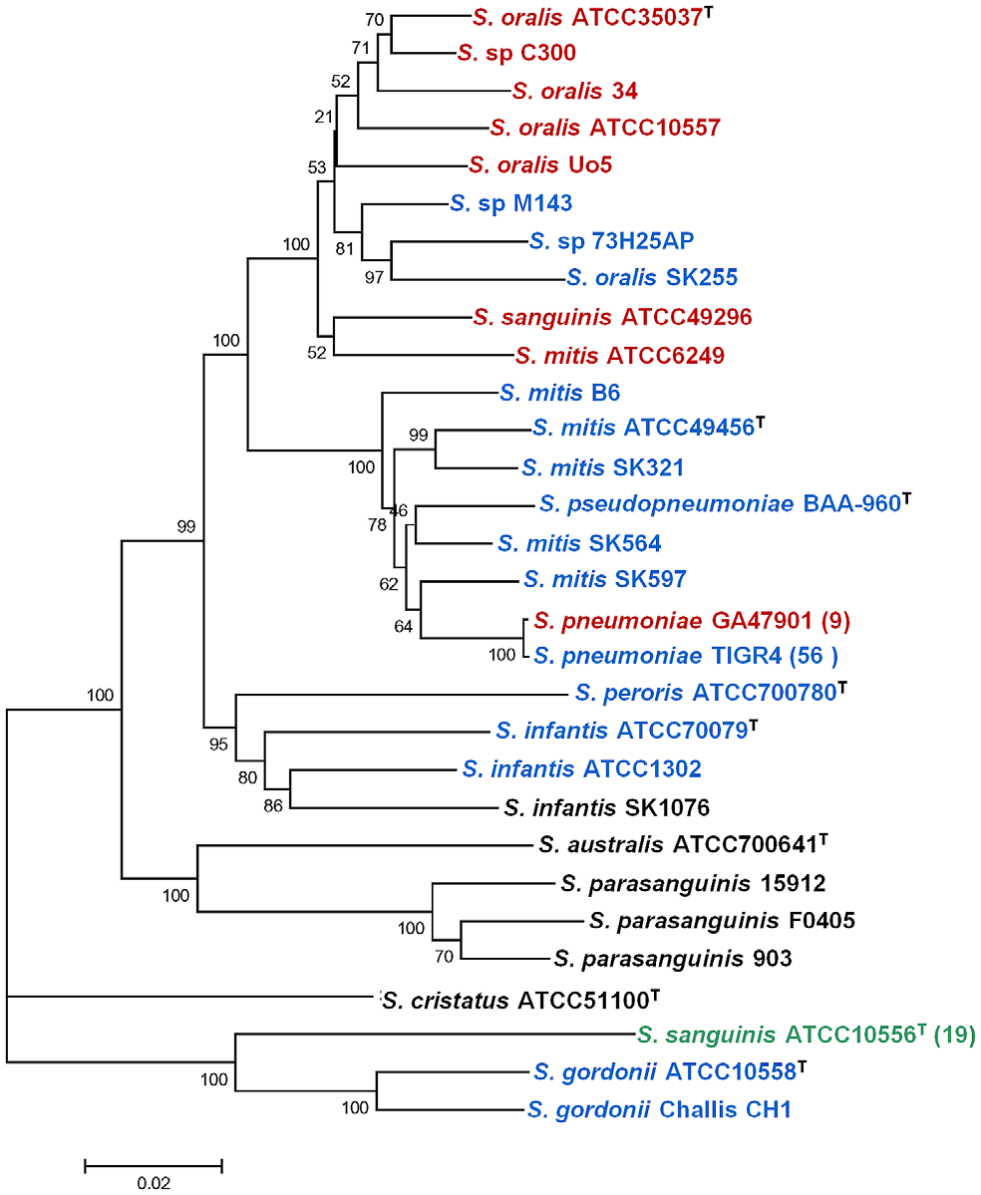
Distribution of pilus islet PI-2 and conservation of the *pepT-hemH* region in Mitis group streptococci. Phylogenetic tree constructed with the neighbor-joining method in MEGA version 5.0 based on electronic Multilocus Sequence Analysis (eMLSA) of Mitis group streptococci. Bootstrap values (%) are based on 500 replications. Strains include the strains used in this study and all Mitis group streptococci with partial or complete genomic sequence deposited at the National Center for Biotechnology Information (NCBI). Type strains are indicated ^(T)^, and for species with >5 sequenced strains the number of additional strains with a particular *pepT-hemH* genotype is indicated in brackets. Strains containing a linked *pepT*-*hemH* region (blue), a PI-2-encoding pilus islet integrated in the *pepT-hemH* region (red) or a PI-2-unrelated insertion in the *pepT-hemH* region (green) are highlighted. Strains that lack a *pepT-hemH* region are shown in black. GenBank accession numbers are: *S. oralis* ATCC35037: AEDW0000000; *S.* sp. C300: ACRJ00000000; *S. oralis* Uo5: FR720602; *S.* sp. M143: ACRK00000000; *S.* sp. 73H25AP: AEEP00000000; *S. oralis* SK255: AFNM00000000; *S. sanguinis* ATCC49296: AEPO00000000; *S. mitis* ATCC6249: AEEN00000000; *S. mitis* B6: FN568063; *S. mitis* ATCC49456 ( = NTCT12261): AEDX00000000; *S. mitis* SK321: AEDT00000000; *S. mitis* SK564: AEDU00000000; *S. mitis* SK597: AEDV00000000; *S. pneumoniae* GA47901: AFGR00000000; *S. pneumoniae* TIGR4: AE005672; *Streptococcus peroris* ATCC700780: AEVD00000000; *Streptococcus infantis* ATCC70079: AEVD00000000; *S. infantis* ATCC1302: AEDY00000000; *S. infantis* SK1076: AFNN00000000; *S. australis* ATCC700641: AEQR00000000; *S. parasanguinis* 15912: CP002843; *S. parasanguinis* F0405: AEKM00000000; *S. parasanguinis* 903: AEVE00000000; *S. cristatus* ATCC51100: AFAZ00000000; *S. sanguinis* ATCC10556 ( = SK1): AFAZ00000000; *S. gordonii* Challis CH1: CP000725. The scale bar refers to genetic divergence as calculated by the MEGA5 software.

PI-2 pilus encoding islets have been identified in strains of four different species: *S. pneumoniae*, *S. oralis, S. mitis and S. sanguinis,* and also in the unassigned streptococcal strain C300, and the islet was always inserted between the *pepT-hemH* genes (Figure 1; [21], [33]). To determine the phylogenetic relationship between the PI-2-containing strains identified in this study and the extent of conservation of the *pepT-hemH* region throughout the Mitis group streptococci electronic Multilocus Sequence Analysis (eMLSA, [46]) was performed. eMLSA is based on seven independent genetic loci, selected to reflect the phylogenetic relationship among viridans streptococci [46]. The analysis included all strains of species of Mitis group streptococci with currently available genomic sequences (deposited as complete or partial genomic sequences at the National Center for Biotechnology [NCBI]) and the additional strains used in this study. Figure 4 shows that PI-2-containing oral streptococci all belonged to a cluster of strains that included the *S. oralis* type strain ATCC35037. The identification of *S. mitis* strain ATCC6249 within this cluster of mostly *S. oralis* strains is consistent with earlier reports describing *S. mitis* strains that were more closely related to *S. oralis* strains than to other *S. mitis* strains, disclosing *S. mitis* as a very heterogenous species among streptococci [6]. Surprisingly, also *S. sanguinis* ATCC49296 was more closely related to strains of *S. oralis* than to *S. sanguinis* (Figure 4). This finding suggests that *S. sanguinis* ATCC49296 has been misclassified as *S. sanguinis*. Hence, all PI-2-containing oral streptococcal strains are closely related to *S. oralis* strains.

The *pepT-hemH* region was conserved throughout most of the species of Mitis group streptococci, including the important human pathogen *S. pneumoniae* and even the more distantly related *Streptococcus gordonii* (Figure 4). The sequence identity of the approximately 150-bp intergenic region between *pepT* and *hemH* ranged from >52% for *S. gordonii* to 67–96% for all other species, and between 76–92% and 72–98% for the flanking genes *pepT* and *hemH*, respectively, relative to *S. oralis* SK255. The exceptions were *Streptococcus parasanguinis* (formerly *S. parasanguis*), the close relative of *S*. *parasanguinis, Streptococcus australis* [47], and *Streptococcus cristatus* (formerly *S. crista*) and an isolate of *Streptococcus infantis* (Figure 4). In these species or strains, respectively, unrelated putative proteins are encoded adjacent to *pepT*, without conservation of the intergenic region, and *hemH* homologs, if present, were encoded in a distant region of the genome, likely excluding these strains from acquisition of PI-2 at this site. In all *S. sanguinis* strains (except the above discussed ATCC49296, supporting its misclassification) a 4.1 kb region, which is unrelated to PI-2, was inserted between *pepT* and *hemH* (Figure 1). This finding demonstrates that the *pepT*-*hemH* insertion site is a hotspot of recombination and not specific for PI-2.

Bagnoli *et al*. [21] described a 7-bp sequence (TCCTTTT) putative insertion site for PI-2, which is duplicated in some of the pneumococcal PI2-encoding strains. However, this duplicated sequence was absent from the borders of the PI-2 islets in the oral streptococci, suggesting acquisition of PI-2 by homologous recombination within the 150-bp intergenic region between *pepT* and *hemH* or the flanking genes. The conservation of the *pepT-hemH* region among most of the Mitis group streptococci provides the possibility for PI-2 acquisition by other strains of the Mitis group streptococci. PI-2 was not identified in any other chromosomal location.

Taken together, PI-2 pilus encoding islets were identified in strains of *S. oralis* and *S. mitis* and the insertion site between *pepT* and *hemH* was conserved among most species of Mitis group streptococci. The expressed PI-2 pili were composed of a strain-specific combination of PitA and PitB variants, which results from intra- and interspecies horizontal gene transfer.

## Discussion

This is the first report of the identification of PI-2 pili in oral streptococci of the Mitis group. PI-2 pili were identified in strains of *S. oralis* and *S. mitis*, species that can be associated with a broad range of pathogenesis traits. These traits lead to early biofilm formation in dental plaque development, adhesion to heart valves resulting in bacterial endocarditis; and involve intra- and interspecies interactions, as well as interactions with host structures and tissues, such as salivary proteins or platelets in the genesis of endocarditis. Adhesion and colonization, have been described for strains of the Mitis group streptococci [48], [49], but the molecular basis for these events is not fully understood and in other Gram positive bacteria, such as actinomyces or enterococci [19], [50], these events are conferred by pili. Thus, PI-2 pili of Mitis group streptococci are candidates for contributing to adhesive interactions.

PI-2 pili have formerly been identified in *S. pneumoniae*. However, pneumococcal PI-2 pili are composed solely of the pilus backbone protein PitB [21]. In contrast, in oral Mitis group streptococci, PI-2 pili were composed of PitB and PitA (the latter is encoded as a pseudogene in *S. pneumoniae)*. PitA is a putative adhesin, which is suggested by the presence of a vWA domain. The vWA domain is present in both PitA variants, such as in ATCC35037 and ATCC6249 despite their 20% sequence variation. Further, the second sortase gene encoded in the PI-2 pilus islet, *srtG2,* also a pseudogene in most pneumococci [21], is predicted to be functional in all the analyzed oral streptococcal PI-2 islets. Thus, PI-2 pili in oral Mitis group streptococci retained features that have been lost in the pneumococcal PI-2 pilus islet.

Analysis of the PI-2 islets provided evidence for horizontal gene transfer (HGT) events, which were not limited to the acquisition of the entire PI-2 islet but also involved recombination events within the islet, as evident by the presence of combinations of particular PitA or PitB variants found within and beyond species (Figure 2). The observed sequence variation between the *pitA* and *pitB* variants, respectively, is consistent with the variation in previously identified antibiotic resistance-conferring mosaic genes in penicillin binding proteins (PBPs) of *S. pneumoniae* that originated from PBPs of *S. mitis* or *S. oralis* [51], [52], [53] and mosaic blocks in the virulence factor NanA [54], with a typical difference between 20%–30% [51], [52]. Western blot and immunogold-labeling EM revealed that the PitB variant of ATCC35037/ATCC6249 had different antigenic properties than ATCC10557 and ATCC49296/Uo5/C300, respectively. Hence, the 20%–25% sequence variation between the PitB variants was sufficient to abolish polyclonal antibody cross-reactivity. PI-2 pili sequence variation may contribute to host evasion or specific pathogenic events and has implications for pilus proteins as vaccine candidates.

The modular structure of the pilus proteins, with highly conserved signal sequences and motifs in the cell wall sorting signal, in combination with conserved biosynthetic genes, provides a framework to incorporate variable domains that may result in pili with different functions. The PitA variants as well as the PitB variants may facilitate adhesion. The PitB-based PI-2 pilus in *S. pneumoniae* mediates adhesion to eukaryotic cells, although with far less efficiency than observed for other Gram-positive pili [21] that employ the accessory pilus adhesins for this interaction. The putative adhesive vWA domain in PitA may mediate binding to various substrates. Konto-Ghiorghi *et al*. have shown that the vWA domain in the pilus protein Gbs1474 (PilC) of *S. agalactiae* is critical for adherence to epithelial cells [27]. In addition, Gbs1474 has a vWA-domain independent function in biofilm formation [27]. Dissection of the functional role of streptococcal PI-2 pili will need to consider the role of both pilus proteins and also address the broad spectrum of bacteria-bacteria as well as bacteria-host adhesive interactions associated with the Mitis group streptococci.

A striking feature in the *S. pneumoniae* PI-2 islet is the inactivation of *pitA* and *srtG2*. In general, gene inactivation was not observed in the streptococcal PI-2 gene clusters. But the *pitB* gene of *S. oralis* strain 34 lacked a start codon, and pilus structures were not seen. However, PitA monomer was identified in cell wall extracts of this strain and may exert an adhesive function on the bacterial surface, even though not as part of a pilus structure. Also, the absence of PitA in strain ATCC6249 requires further evaluation. Further studies will have to determine whether selective gene inactivation but retention of parts of the biosynthetic machinery of the PI-2 pili may provide an additional molecular mechanism to enhance variation in structure and function of PI-2 pili, and whether this may modulate the tissue tropism of certain strains.

Identification of the PI-2 islet in species of *S. oralis*, *S. mitis* and the more distantly related *S. sanguinis* suggested a distribution of PI-2 throughout the entire streptococcus Mitis group. However, eMLSA revealed that all PI-2-containing strains were closely related to each other and to PI-2 lacking strains of *S. oralis*. This included *S.* sa*nguinis* ATCC49296, which was in fact identified as *S. oralis* based on biochemical characterization in an earlier study [55], and based on eMLSA should be considered misclassified as *S. sanguinis*. Although HGT and the ability to develop competence for genetic transformation [56], [57], [58] has been observed throughout the Mitis group of streptococci, the increasing number of available genomic sequences has revealed that HGT is a particularly common theme among *S. pneumoniae*, *S. mitis* and *S. oralis* [7]. The identified distribution of PI-2 islets suggests that they are part of a common gene pool shared by *S. pneumoniae, S. mitis,* and *S. oralis*.

Filamentous surface structures have long been observed on the surface of oral streptococci, including strains of *S. oralis*, *S. mitis* and *S. sanguinis* (most of them then classified as *S. sanguis*) by electron microscopy [59], [60], [61], [62], [63]. They were visualized by negative staining and described as tufts, fibrils or fimbriae, and ranged from a 30 nm to ≤700 nm that either coexisted on the bacterial cell or seemed to be mutually exclusive [60], [63]. *S. oralis* strains ATCC35037, ATCC10557, and 34 were described to express fibrils, but no fimbriae [64]. The molecular nature of these structures remained elusive until cross-reactive antibodies against the Csh-like protein, first identified in *S. gordonii* [25], detected fibrils of 50–80 nm on several *S. oralis* and *S. sanguinis* strains [64]. CshA homologs are encoded in *S. oralis* and *S. mitis*, but due to major sequence variation in the binding domain, their function remains to be determined [64]. However, the PI-2 pili described in this study were considerably longer (>700 nm), and therefore inconsistent with these fibrilar surface structures. The failure to identify pili in earlier studies is consistent with our inability to detect pili by negative staining alone. In fact, the lack of sufficient contrast of Gram-positive pili by negative staining has contributed to the delay in identification of Gram-positive pili [10]. Several previous studies [60], [62], [63] have tried to correlate the observed surface structures on Mitis group streptococci with pathogenesis relevant traits, such as coaggregation and binding to salivary proteins [60], [62], [63], but the results have been inconclusive. Dissection of the role of PI-2 pili may help to elucidate some of these events.

Taken together, genetically variable PI-2 pili were identified and characterized in multiple strains of *S. oralis and S. mitis*. The observed intra- and interspecies variation in the genes encoding these pili provide evidence for horizontal gene transfer events between and within PI-2 islets of these species. Further work will have to determine whether the pilus protein variants confer different adhesive properties to the respective strain. The discovery of PI-2 pili in oral Mitis group streptococci may provide a key to dissect the heterogeneity of interspecies and host interactions associated with these bacteria.

## Materials and Methods

### Bacterial strains and growth conditions

All streptococcal strains used in this study are listed in Table 1. Streptococcal strains were routinely grown in Todd-Hewitt broth (BD) supplemented with 0.5% yeast extract (THY) and antibiotics, if appropriate. Antibiotics were used in the following concentrations: spectinomycin 110 µg/ml; kanamycin 50 µg/ml for *Escherichia coli* and 800 µg/ml for streptococcal strains.

### PCR-based screen for PI-2

To identify PI-2 islets primers sipA_so_up and sipA_so_dn (Table S1), designed against the PI-2 specific gene *sipA,* [21] were used. To determine the absence of PI-2 from the described insertion site primers pepT_so_F and hemH_so_R (Table S1) were designed against the flanking genes *pepT* and *hemH*. All primers were designed based on the sequence of *S. oralis* strain ATCC35037.

### Electronic Multilocus Sequence Analysis (eMLSA)

The phylogenetic relationship between strains used in this study was determined by multilocus sequence analysis [46] following the instructions provided online for the viridans streptococci project (www.viridans.emlsa.net). Multilocus sequence information of strains from genomic sequencing projects was retrieved from the according loci of the genomic sequences, in case the data was not already included in the eMLSA database, and from strains with not yet available sequence information by amplification and sequencing of the corresponding loci. Primers and conditions were used according to the online instructions. Alignment of concatenated sequences was performed with MEGA version 5.0 [65].

### Construction of PI-2 deletion mutants

3′- and 5′-flanking regions of PI-2 of *S. oralis* strain ATCC35037 were amplified using primer pairs pepT_so_F/PitA_so_3 and SrtG2_2/hemH_so_R, respectively (Table S1). Both PCR products were used in a PCR based overlap-extension reaction [66], introducing a *Mfe*I restriction site between the two fused fragments. The PCR product was cloned into the pCR-8/GW-TOPO vector (Invitrogen), resulting in pCR8-ΔPI-2. The kanamycin-cassette of pSF151 [67] was amplified using primers KM_F and KM_R (Table S1), cloned into the TOPO vector pCR2.1 (Invitrogen), recovered by *Eco*RI digestion, and ligated into *Mfe*I-digested pCR8-ΔPI-2. The resulting plasmid pCR-ΔPI-2::km was used to transform strains ATCC35037 and ATCC10557 following established protocols [68]. Competence stimulating peptide specific for ATCC35037 and ATCC10557 (synthesized by Genscript Corp.) was used for transformation [58], [69].

### Production of recombinant PitA and PitB

The regions of *pitA* and *pitB*, corresponding to the mature proteins (bp 106 to bp 2484 and bp 91 to bp 1128, respectively) were amplified from chromosomal DNA of *S. oralis* ATCC35037 using primer pairs PitA_so_SmaI_F/PitA_so_NotI_R, and PitB_so_BamHI_F/PitB_so_Xho_R, respectively (Table S1) and cloned into the *E. coli* expression vector pET-32a+ (Stratagene). The thioredoxin/6xHis-fusion proteins were purified using the B-PER 6xHis Fusion Purification Kit (Pierce). The N-terminal thioredoxin/6xhis-tag was removed by cleavage with recombinant enterokinase (Novagen) according to the manufacturer's recommendations. Purified PitA and PitB proteins were used to produce polyclonal antisera in rabbits (Covance Research Products Inc.).

### Preparation of streptococcal cell wall extracts

Cell wall extracts of streptococcal strains were prepared from bacteria grown on trypticase soy agar (TSA) plates containing 5% sheep blood (BD) overnight at 37°C and 5% CO_2_. Bacteria were collected from plates by adding 750 µl 10 mM Tris, pH 8 buffer to the plate surface, followed by resuspension and recovery of the bacteria. Samples were centrifuged for 5 min at 13000 x g and bacterial pellets were used for further processing. Bacterial pellets (approximately 40 mg per strain) were resuspended in 250 µl 10 mM Tris; pH 8.0 supplemented with 400 U/ml mutanolysin (Sigma-Aldrich, St. Louis, MO, USA) and 30% raffinose. After 2 h of incubation at 37°C, cell wall extracts were collected by centrifugation at 12,000 × *g* for 15 min. 25 µl cell wall extract were mixed with sodium dodecyl sulfate–polyacrylamide gel electrophoresis (SDS-PAGE) sample buffer and boiled for 10 min immediately before separation on a 3%–8% Tris-Acetate polyacrylamide gel (NuPAGE; Invitrogen). For immunostaining, proteins were transferred to a nitrocellulose membrane and detected with polyclonal anti-PitA (1:50,000 diluted) or anti-PitB antiserum (1∶20,000).

### Negative staining and immunogold electron microscopy

Strains were grown on TSA-blood agar plates (5%-sheep blood, BD) overnight at 37°C and 5% CO_2._ Bacteria were resuspended in PBS before being applied to carbon-coated copper grids (300 mesh, EMS), and stained with 1% methylamine tungstate for 30 seconds. Alternatively, strains were applied to carbon-coated nickel grids (300 mesh, EMS) and immunostained according to established protocols [21], [70]. Anti-PitA and anti-PitB antiserum were used at a 1∶10,000 and a 1∶4,000 dilution, respectively, and a 10 nm-gold-conjugated goat anti-mouse IgG was used as secondary antibody at 1∶20 dilution. Following the immunogold staining, the grids were negative stained as described above and analyzed using a Hitachi H-7500 transmission electron microscope.

### Nucleotide sequence accession numbers

The following GenBank accession numbers were assigned to PI-2 of *S .oralis* ATCC10557: JF496566 and *S. oralis* 34: JF496567.

## Supporting Information

Figure S1 **Alignment of PitB proteins of oral Mitis group streptococci and** ***S. pneumoniae.*** **PitBs of** ***S. oralis*** **Uo5 and** ***S*** **. sp.** C300 are not shown (identical to PitB of *S. sanguinis* ATCC49296). Only residues different from the consensus sequence are indicated. Sequence positions based on the *S. oralis* ATCC35037 sequence are indicated above the alignment. The Signal peptides (SP) and cell wall sorting signals (CWSS) are underlined and the LPXTG-like motif (VTPTG) is shown in bold. Signal peptides were predicted by the SignalP program. Conserved amino acids involved in intramolecular isopeptide bonds in *S. pneumoniae* PitB are indicated by an asterisk followed by the number for bond 1 and 2, respectively. GenBank accession numbers are as in Fig. 1. Alignment was performed with ClustalW software.
(PPT)

Figure S2 **Evidence for past recombination events in PI-2 pilus islets of Mitis group streptococci.** A. Schematic overview of PI-2 pilus islets. Sequences of *S. oralis* Uo5 and *S*. sp. C300 are not shown (almost identical to *S. sanguinis* ATCC49296). Gene regions encoding conserved motifs are indicated by a black bar below the gene. SP: Signal peptide, vWA: von Willebrand A domain, CWSS: cell wall sorting signal. Red squares indicate the regions of sudden, major changes in sequence polymorphisms aligned in panels B.-E. In panels B.-E. Regions of major sequence polymorphisms are indicated in grey. GenBank accession numbers: *S. oralis* strains ATCC35037: AEDW01000020; ATCC10557: JF496566; and 34: JF496567; *S. mitis* ATCC6249: AEEN01000012; *S. sanguinis* ATCC49296: AEPO01000013; and *S. pneumoniae* GA47884: GU256423. Alignment was performed with ClustalW software.
(PPT)

Table S1 **Primers used in this study.**
(DOC)
