# Supplementary material for: Mitis Group Streptococci Express Variable Pilus Islet 2 Pili
Source: PLoS One. 2011 Sep 22;6(9):e25124. doi: 10.1371/journal.pone.0025124 (PMC3178606; doi:10.1371/journal.pone.0025124)
Supplement: Figure S1 — Alignment of PitB proteins of oral Mitis group streptococci and S. pneumoniae. PitBs of S. oralis Uo5 and S . sp. C300 are not shown (identical to PitB of S. sanguinis ATCC49296). Only residues different from the consensus sequence are indicated. Sequence positions based on the S. oralis ATCC35037 sequence are indicated above the alignment. The Signal peptides (SP) and cell wall sorting signals (CWSS) are underlined and the LPXTG-like motif (VTPTG) is shown in bold. Signal peptides were predicted by the SignalP program. Conserved amino acids involved in intramolecular isopeptide bonds in S. pneumoniae PitB are indicated by an asterisk followed by the number for bond 1 and 2, respectively. GenBank accession numbers are as in Fig. 1. Alignment was performed with ClustalW software. (PPT) [file pone.0025124.s001.ppt]

## Slide 1
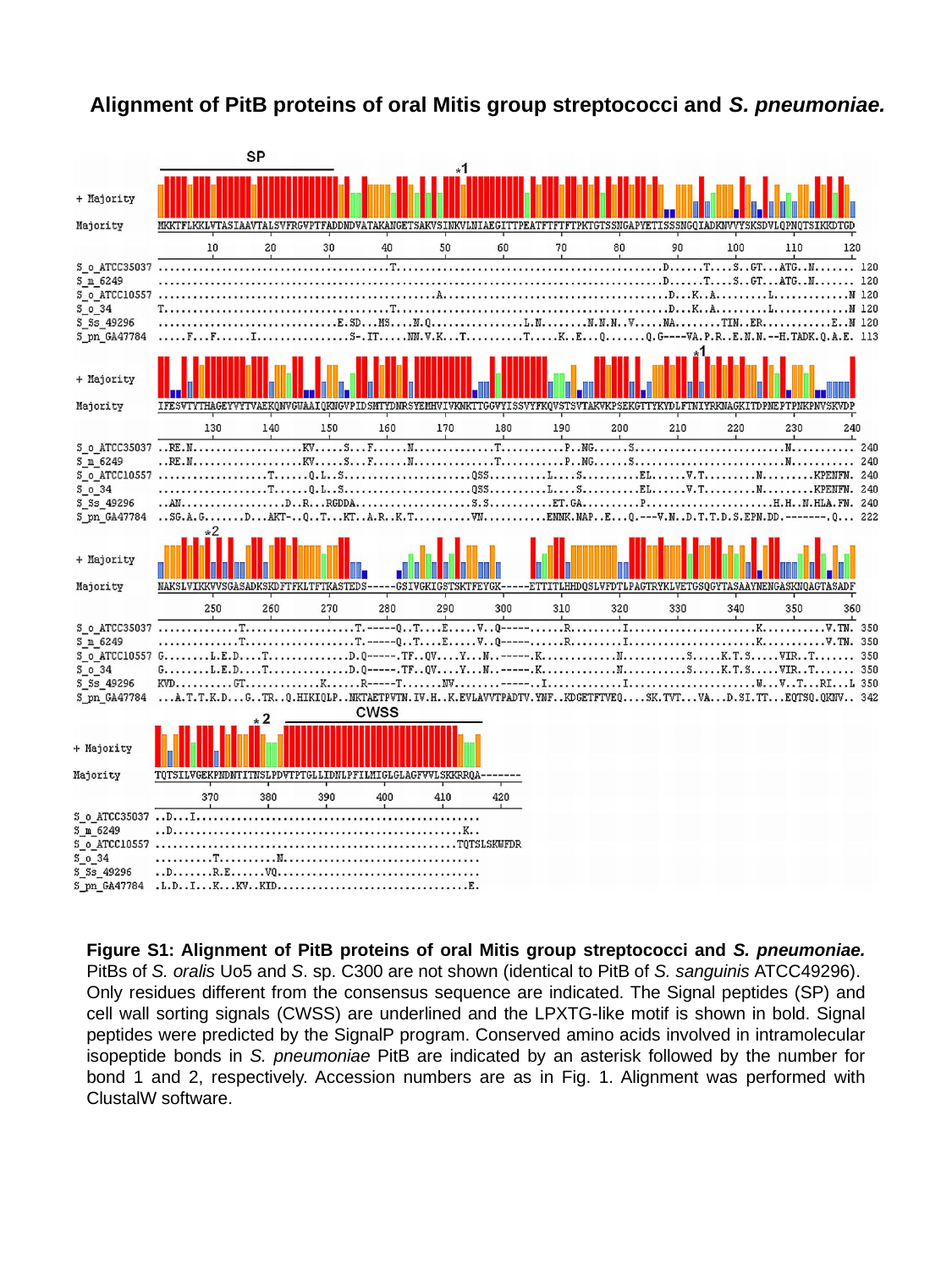

Alignment of PitB proteins of oral Mitis group streptococci and S. pneumoniae.
Figure S1: Alignment of PitB proteins of oral Mitis group streptococci and S. pneumoniae. PitBs of S. oralis Uo5 and S. sp. C300 are not shown (identical to PitB of S. sanguinis ATCC49296). Only residues different from the consensus sequence are indicated. The Signal peptides (SP) and cell wall sorting signals (CWSS) are underlined and the LPXTG-like motif is shown in bold. Signal peptides were predicted by the SignalP program. Conserved amino acids involved in intramolecular isopeptide bonds in S. pneumoniae PitB are indicated by an asterisk followed by the number for bond 1 and 2, respectively. Accession numbers are as in Fig. 1. Alignment was performed with ClustalW software.
