# Supplementary material for: Mitis Group Streptococci Express Variable Pilus Islet 2 Pili
Source: PLoS One. 2011 Sep 22;6(9):e25124. doi: 10.1371/journal.pone.0025124 (PMC3178606; doi:10.1371/journal.pone.0025124)
Supplement: Figure S2 — Evidence for past recombination events in PI-2 pilus islets of Mitis group streptococci. A. Schematic overview of PI-2 pilus islets. Sequences of S. oralis Uo5 and S. sp. C300 are not shown (almost identical to S. sanguinis ATCC49296). Gene regions encoding conserved motifs are indicated by a black bar below the gene. SP: Signal peptide, vWA: von Willebrand A domain, CWSS: cell wall sorting signal. Red squares indicate the regions of sudden, major changes in sequence polymorphisms aligned in panels B.-E. In panels B.-E. Regions of major sequence polymorphisms are indicated in grey. GenBank accession numbers: S. oralis strains ATCC35037: AEDW01000020; ATCC10557: JF496566; and 34: JF496567; S. mitis ATCC6249: AEEN01000012; S. sanguinis ATCC49296: AEPO01000013; and S. pneumoniae GA47884: GU256423. Alignment was performed with ClustalW software. (PPT) [file pone.0025124.s002.ppt]

## Slide 1
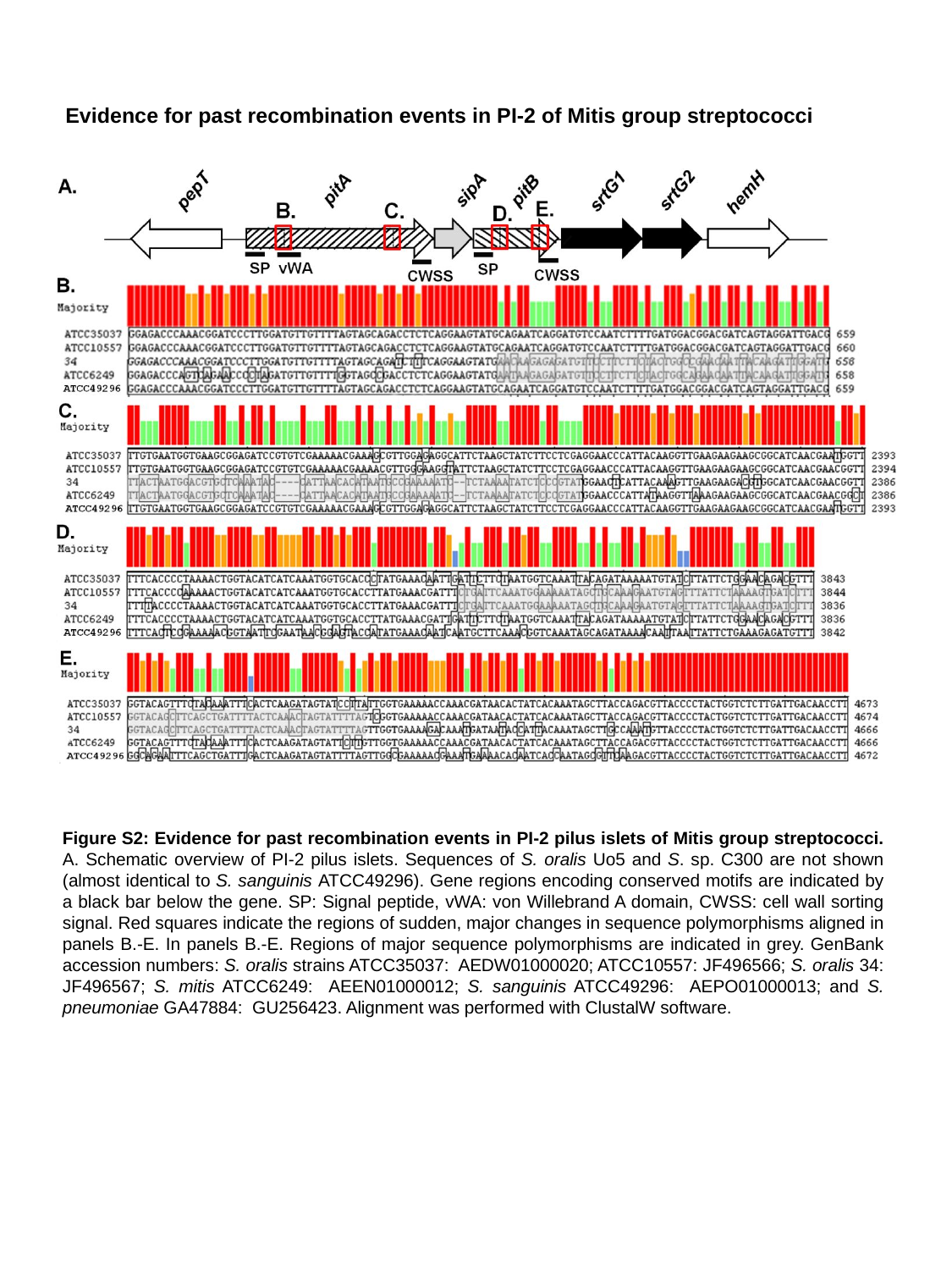

Evidence for past recombination events in PI-2 of Mitis group streptococci
Figure S2: Evidence for past recombination events in PI-2 pilus islets of Mitis group streptococci. A. Schematic overview of PI-2 pilus islets. Sequences of S. oralis Uo5 and S. sp. C300 are not shown (almost identical to S. sanguinis ATCC49296). Gene regions encoding conserved motifs are indicated by a black bar below the gene. SP: Signal peptide, vWA: von Willebrand A domain, CWSS: cell wall sorting signal. Red squares indicate the regions of sudden, major changes in sequence polymorphisms aligned in panels B.-E. In panels B.-E. Regions of major sequence polymorphisms are indicated in grey. GenBank accession numbers: S. oralis strains ATCC35037: AEDW01000020; ATCC10557: JF496566; S. oralis 34: JF496567; S. mitis ATCC6249: AEEN01000012; S. sanguinis ATCC49296: AEPO01000013; and S. pneumoniae GA47884: GU256423. Alignment was performed with ClustalW software.
