# Supplementary material for: Mitis Group Streptococci Express Variable Pilus Islet 2 Pili
Source: PLoS One. 2011 Sep 22;6(9):e25124. doi: 10.1371/journal.pone.0025124 (PMC3178606; doi:10.1371/journal.pone.0025124)
Supplement: Table S1 — Primers used in this study. (DOC) [file pone.0025124.s003.doc]

Table S1. Primers used in this study ^a^

| Primer | Sequence (5'->3') | |
| --- | --- | --- |
| KM_F | GTATTTAAGGTTTTAGAATGCAAGGAAC | |
| KM_R | GGTACTAAAACAATTCATCCAGTAAAA | |
| sipA_so_up | AGGAATAATCCTTCCTCTAGGAGG | |
| sipA_so_dn | GAGGACAAAGAGTTGTCCTGCTGG | |
| pepT_so_F | GGAGTAGTAGTAGAGTGTTCATC | |
| PitA_so_3 | g**caattg**cccgggcctagTAACAGTGTAACTAAACGGCGTGC | |
| SrtG2_2 | ctaggcccggg**caattg**cGAAATAGTAGACGTCGGAAGCG | |
| hemH_so_R | CAATCTCATCTGTCCAGAAGTCC | |
| PitA_so_SmaI_F | **cccggg**GATTCTACTACAGAACCTCAGACAAC | |
| PitA_so_NotI_R | **gcggccgc**TCAGGTTTCAGGTACACTGTTCTTATTATTCG | |
| PitB_so_BamHI_F | **ggatcc**GATGATAATGATGTGGCGACTGCC | |
| PitB_so_Xho_R | **ctcgag**TCAAGTAGGGGTAACGTCTGGTAAGC | |
|  | |  |

^a^ Introduced restriction sites are indicated in bold. Uppercase letter represent bases complementary to streptococcal sequence. Lowercase letters represent bases added to facilitate overlap-PCR or cloning.
